# Supplementary material for: Acceptability of Medical Male Circumcision as an HIV Prevention Intervention among Male Learners in a South African High School
Source: Healthcare (Basel). 2024 Jul 6;12(13):1350. doi: 10.3390/healthcare12131350 (PMC11241183; doi:10.3390/healthcare12131350)
Supplement: Supplementary file 1 [file healthcare-12-01350-s001.zip › healthcare-3055907-supplementary.pdf]

ANNEXURES

Annexure S1: Questionnaire

A STUDY TO INVESTIGATE KNOWLEDGE AND ACCEPTABILITY OF MALE MEDICAL CIRCUMCISION AS AN HIV PREVENTION INTERVENTION AMONGST GRADE 11 AND 12 BOYS AT SEHUSHE HIGH SCHOOL IN LIBODE.

Questionnaire

1. **Age** ……………………...

2. **Grade** ……………………

3. **Religion (Tick one)**

Christian	
Hindu	
Rastafarian	
No Christian	
Other	

4. **Place of Residence (Tick one)**

Urban area	
Rural area	
Informal Settlement	

5. **What is the current marital status of your parents? (Tick one)**

Married/living together	
Single	
Separated	
Divorce	
Widowed	

6. **What is the current employment status of your parents? (Tick all the relevant answers)**

Father formally employed	
Mother formally employed	
Father self-employed	
Mother self-employed	
I don’t know about my father	
I don’t know about my mother	
Father deceased	
Mother deceased	

7. **Who do you currently live with? (Tick all the relevant answers)**

Father	
Mother	
Brother	
Sister	
Grandfather	
Grandmother	
Uncle	
Aunty	
Other relative	
Other (specify)	

8. **Knowledge about Medical Male Circumcision**

Is there any difference between Medical Male Circumcision and Traditional Male Circumcision?	Yes	No	I don't know
Is Medical Male Circumcision safe when it is done accordingly?	Yes	No	I don't know
Is Medical Male Circumcision done at the clinic?	Yes	No	I don't know
Is Medical Male Circumcision done at the hospital?	Yes	No	I don't know
Is Medical Male Circumcision done by a nurse?	Yes	No	I don't know
Is Medical Male Circumcision done by a doctor?	Yes	No	I don't know
Is Medical Male Circumcision done by a traditional surgeon (ingcibi)?	Yes	No	I don't know
Is Medical Male Circumcision done by any circumcised men?	Yes	No	I don't know
Does Medical Male Circumcision reduce the risk of STI among males?	Yes	No	I don't know
Does Medical Male Circumcision reduce the risk of STI among females?	Yes	No	I don't know
Does Medical Male Circumcision reduce the risk of HIV infection among males?	Yes	No	I don't know
Does Medical Male Circumcision reduce the risk of HIV infection among females?	Yes	No	I don't know
Does Medical Male Circumcision prevent HIV infection entirely among males?	Yes	No	I don't know
Does Medical Male Circumcision prevent HIV infection entirely among females?	Yes	No	I don't know

9. **Can you tell me all the places you have heard about Medical Male Circumcision? (Tick all relevant)**

Never heard about it. **SKIP to question 10**	
Television	
Radio	
Facebook	
Twitter	
Posters / Billboards	
Pamphlets / Booklets / Hand outs	
Community events	
Friend / Relative	
From and HIV&AIDS organisation	
Clinic / Hospital	
Newspaper	
Don’t remember	
Other (specify)	

10. **Acceptability of Medical Male Circumcision**

Would you consider undergoing Medical Male Circumcision as an HIV prevention intervention?	Strongly agree	Agree	Unsure	Disagree	Strongly disagree
Do you think your parents would allow you to be medically circumcised for HIV prevention intervention?	Strongly agree	Agree	Unsure	Disagree	Strongly disagree
Do you think most of your family members would allow you to be medically circumcised for HIV prevention intervention?	Strongly agree	Agree	Unsure	Disagree	Strongly disagree
Can most of your male friends allow you to be medically circumcised for HIV prevention intervention?	Strongly agree	Agree	Unsure	Disagree	Strongly disagree
Can most of female friends allow you to be medically circumcised for HIV prevention intervention?	Strongly agree	Agree	Unsure	Disagree	Strongly disagree
Can most of your male schoolmates allow you to be medically circumcised for HIV prevention intervention?	Strongly agree	Agree	Unsure	Disagree	Strongly disagree
Can most of female schoolmates allow you to be medically circumcised for HIV prevention intervention?	Strongly agree	Agree	Unsure	Disagree	Strongly disagree
Can most of your community members allow you to be medically circumcised for HIV prevention intervention?	Strongly agree	Agree	Unsure	Disagree	Strongly disagree

11. **Which type of circumcision do you prefer? (Tick one)**

Medical Male Circumcision	
Traditional Male Circumcision	

12. **Would you refer a friend for MMC? (Tick one)**

Yes	
No	

13. **Would you refer a relative for MMC? (Tick one)**

Yes	
No	

14. **Are there any complications related to Medical Male Circumcision?**

Yes	
No	

15. **If yes, which one of the following? (Tick all relevant)**

Continuous excessive bleeding	
Severe pains of the penis	
Infections (Urinary Tract Infection-UTI)	
Wound not healed at 60 days after surgery	
Partial amputation	

!!!!!!!!!!………..Thank you very much for participating in this study………
